# Validation of 24‐h dietary recall for estimating nutrient intakes and adequacy in adolescents in Burkina Faso

**DOI:** 10.1111/mcn.13014

**Published:** 2020-04-26

**Authors:** Joanne E. Arsenault, Mourad Moursi, Deanna K. Olney, Elodie Becquey, Rasmané Ganaba

**Affiliations:** ^1^ Institute for Global Nutrition University of California Davis California USA; ^2^ Intake, Center for Dietary Assessment FHI Solutions Washington DC USA; ^3^ Poverty, Health, and Nutrition Division International Food Policy Research Institute Washington DC USA; ^4^ AFRICSanté Bobo‐Dioulasso Burkina Faso

**Keywords:** adolescent, childhood diet, dietary assessment tools, dietary intake assessment, dietary methods, low income countries, validation

## Abstract

Data on dietary nutrient intakes of adolescents in low‐ and middle‐income countries (LMIC) is lacking partly due to the absence of validation studies of the 24‐h recall method in adolescents. We conducted a validation study of 24‐h recall (24HR) compared with observed weighed records (OWR) in adolescents (*n* = 132, 10–11 years; *n* = 105, 12–14 years). Dietary data were collected for the same day by both methods by conducting the 24HR the day after the OWR. For OWR, all foods consumed by adolescents from the first to last meal of the day were weighed; for 24HR adolescents reported foods consumed using portion aids. Food intakes were converted to nutrients. Nutrient intakes by both methods were tested for equivalence by comparing the ratios (24HR/OWR) with equivalence margins of within ±10%, 15% and 20% of the ratio. Prevalences of inadequacy (POIs) were obtained using the NCI method. Mean ratios for energy were 0.88 and 0.92, for younger and older adolescents, respectively, and other nutrients ranged between 0.84 and 1.02. Energy intakes were equivalent within the 15% bound, and most nutrients fell within the 20% bound. POI was overestimated by 24HR, but differences were less than 25 percentage points for most nutrients. Half of adolescents omitted foods in recalls, mainly sweet or savoury snacks, fruits and beverages. Our study showed that adolescents underestimated intakes by 24HR; however, the degree of underestimation was generally acceptable for 12–14‐year‐olds within a bound of 15%. Errors could possibly be reduced with further training and targeted probing.

**Key messages**

- Adolescents underestimate their food intake by 24‐h recall, resulting in underestimation of nutrient intake and overestimation of inadequacy.
- The degree of underestimation was generally acceptable for 12–14‐year‐olds within an equivalence bound of about ±280 kcal/day (15%), which is similar to or even better than some validations of adults for which the 24‐h recall method is widely used for research and surveillance purposes.
- Based on these results, the 24‐h dietary recall method can be used in adolescents to assess population dietary risk levels, changes in intakes over time and differences between groups.

## 1 INTRODUCTION

The importance of nutrition for adolescents, defined as children aged 10–19 years by WHO, in low‐ and middle‐income countries (LMIC), particularly girls, has been highlighted in recent years. However, data to inform programmes and progress for addressing the health and nutrition of this population group is lacking (Christian & Smith, 2018; Krebs et al., 2017; SPRING, 2018). Dietary data are a key missing piece of information in many LMIC, and particularly for young adolescents, in part, due to a lack of validated tools for this age.

The 24‐h dietary recall method is most commonly used in dietary surveys, and the method has been adapted and validated for use with adults in LMIC to report their own or their young child's intake (Gibson & Ferguson, 2008). The age when children and adolescents can accurately report their food intake without parental assistance is not clear, particularly in LMIC. Having children report their intake without assistance by a parent or caregiver may be logistically easier for studies that are conducted in school settings or during after‐school hours, and the presence of a parent may influence the responses. Although some research from high‐income countries (HIC) has indicated that children between 8 and 12 years can reliably report their food intake, cognitive abilities gradually increase over this time period which may constrain their ability to self‐report before age 12 years (Livingstone & Robson, 2000; Livingstone, Robson, & Wallace, 2004). In Europe, it has been recommended that children up to 14 years should report food intake with parental assistance (Andersen et al., 2011). Although 15 years of age is still considered adolescent by definition, other validations in LMIC adult women have included 15‐year‐olds as the population of interest was women of reproductive age (Alemayehu, Abede, & Gibson, 2011; Ferguson et al., 1995).

The aim of the present study was to validate the 24‐h recall (24HR) method by direct interview, without parental assistance, of 10–11‐ and 12–14‐year‐old adolescents in Burkina Faso against the gold standard of weighed records by full‐day observations. The main objective was to determine if energy and other nutrient intakes were ‘equivalent’ between the two methods, that is, that mean differences between methods fall within acceptable bounds. Additional objectives were to assess sources of error (memory, portion estimation, and use of standard recipes) and to determine micronutrient intake adequacy among Burkinabe adolescents 10–14 years of age by both methods.

## 2 METHODS

### 2.1 Study population and recruitment

The study was conducted in the community of Saaba, a predominately urban district with some rural villages in the Central region of Burkina Faso bordering the capital of Ouagadougou. School attendance in Saaba was approximately 80–85% as reported by the school administration, and the study took place primarily with schools located in the urban part of the district.

We planned to enroll a sample of 108 adolescents ages 10–11 years and 108 ages 12–14 years, with a similar percentage of school children as representative of the study community. The school administrator provided rosters of children from two public and two private schools, and we randomly selected 216 children stratified by two age groups and by school for potential recruitment. In public schools, parents make an annual contribution for each child, but otherwise, the government provides free tuition and a lunch meal to all students. In private schools, parents pay annual tuition fees and an additional annual contribution for each student and a lunch fee if students opt to buy lunch from school. After 85% of the selected children from each school were recruited, we identified children from the same neighbourhoods who were not attending school. Due to some misclassification of ages provided by the schools, an imbalance of children in the two age groups occurred, and our actual sample recruited included 132 10–11‐year‐olds and 105 12–14‐year‐olds.

The intended sample size was based on the ability to detect a 10% difference in energy intake from the two dietary assessment methods, which is an effect size of about 0.33 based on energy intakes of adolescents from another study (Medin, Hansen, Astrup, Ekelund, & Andersen, 2017). However, we wished to also assess whether the two methods were equivalent and recalculated what bound we could detect equivalence given our final sample sizes and actual mean and standard deviation of energy intakes by observation. Our samples were sufficient for detecting equivalence within a 10% bound for the 10–11‐year‐olds and 13% bound for the 12–14‐year‐olds (Sealed Envelope Ltd, 2012). The study was approved by the Ethical Committee of the Ministry of Health of Burkina Faso and the IFPRI Institutional Review Board and was also reviewed by the UC‐Davis IRB with a determination of not requiring their approval.

Recruitment was conducted by visiting identified adolescents at their homes 2–3 days before the intended observation day. Field research assistants (RA) brought an official letter from the school indicating the school's approval for the study and further explained the study and the informed consent procedure. After obtaining written consent by a parent and assent by the child, the RA administered a brief questionnaire to solicit information about when and where the child ate, time of transport to and from school and educational attainment levels of the parents. RA provided the caregiver with a standard size plate and bowl to serve the child to aid in portion size estimation for the 24HRs, however, also emphasized that they should not change their eating habits because we want to know what they eat, and this will help inform future programmes in their community.

### 2.2 Dietary data methods

Twenty RA, comprising of women and men from the study community, were selected from a group of 25 who underwent 7 days of training in the classroom and field. Sixteen conducted the observed weighed records (OWR) and four conducted the 24HRs. One day of dietary data were collected for all children. A second day of dietary data was collected for 62 children by the same OWR RA and by a different 24HR RA that collected the first day.

For OWR, RA arrived at the home at the predetermined time early in the morning before the main morning meal and accompanied the adolescent for the entire day through the last meal of the day. The RA weighed all food and beverages before and after each eating episode using digital scales (7‐kg capacity, MyWeigh KD‐7000) accurate to 1 g. RAs weighed recipe preparations in the homes if they were present in the home during the preparation. When observing recipe preparation was not possible, RA recorded the food description with main distinguishing ingredients. Leftovers that consisted of a mixture of foods that had been weighed separately when served that could not easily be manually separated for weighing were disaggregated by assuming the proportion leftover was the same as the proportion served. The RA also recorded the time, meal, place of consumption and the place of preparation or purchase of each food.

For the 24HR, RA conducted the multiple‐pass interview (Gibson & Ferguson, 2008) on the day after the OWR, usually in the morning or early afternoon when children were on break from school. To aid in portion estimation, the RA used the same type of standard plates (or bowls) that were provided to children. The children were guided to use portion estimation aids that were predetermined for use with specific foods, primarily using actual foods for main staples (rice or *To*—a maize‐based dish), playdough for pieces foods (meat or fish, fruits and bread/sandwiches) and volumetric measures for liquids (sauces, thin porridges and beverages). Preliminary work in the community determined that adolescents could report recipe ingredients but not amounts; therefore, during the study, adolescents were asked to describe main ingredients for mixed dishes/recipes.

A preliminary food list of potential foods and ingredients was prepared prior to the study using information from a prior study conducted in Burkina Faso (Arsenault et al., 2014). Additions were made to the food list during training and throughout the study as needed. Important distinguishing information for food item descriptions were established during training (e.g., type of grains, whether mixed dish contained meat or fish or carrots). Each food item was assigned a unique code for use on paper data collection forms used by the RA.

Household specific recipes were used when observed for OWR only. Standard recipes were used for 24HR or for OWR when a household recipe was not observed. Standard recipes were compiled from averaging the observed recipes from households. School vendor and canteen recipes were observed to create school‐specific standard recipes. For cases when OWR RA was not able to weigh portions of two foods separately or when the child was not able to report portions separately (e.g., rice and leaf sauce), a recipe was compiled based on average proportions of the two foods using available information from the observed OWR. Missing or incomplete data on recipes collected during the study were collected from women in the community after the study.

Conversion factors for the recall methods were collected by the RA during and after the study. Volume equivalents of recipes were collected during observation of recipe preparation in households and were averaged to compile volume to weight conversion factors. Additional conversion factor data were collected from school canteens and vendors for calculation of volume or playdough to food weight conversions. Additional conversion factors were assigned using data from previous studies (Arsenault et al., 2014) and published information (US Department of Agriculture & Agricultural Research Service, 2018; US Department of Agriculture, Agricultural Research Service, & Nutrient Data Laboratory, 2018).

Food composition data were updated from a previous study (Arsenault et al., 2014) using the FAO West African Food Composition Tables (FAO, 2012), with supplemental data from USDA (US Department of Agriculture & Agricultural Research Service, 2018; US Department of Agriculture, Agricultural Research Service, & Nutrient Data Laboratory, 2018) or label information (for fortified foods). Nutrient retention factors were applied to foods that were weighed raw in observed recipes using USDA factors (U.S. Department of Agriculture, 2007). Retention factors for vitamin A of 85% for red palm oil and 95% for fortified vegetable oil used in cooking were based on conservative estimates from the literature given uncertainties about storage and cooking times (Burri, 2012; SUSTAIN, 1998).

### 2.3 Data analysis

Quality checks on paper forms were conducted by the investigator (J.A.) or field supervisor. Data were entered into EpiData databases and checked for errors by double entry. Subsequent cleaning was conducted to determine if appropriate food and measurement method codes were assigned.

Food intakes were converted to nutrient intakes using Stata SE (version 15; StataCorp, College Station, TX) incorporating previously described inputs of amounts recorded, conversion factors, and food composition data. Nutrient intakes were tested for normality using Shapiro–Wilk tests, and all nutrients were log‐transformed for statistical testing. The primary outcome for testing the validity of 24HR was the mean difference in nutrient intakes from the OWR. The difference in log‐transformed nutrient intakes (recall nutrient − observed nutrient) is equivalent to the ratio or proportion (recall nutrient/observed nutrient). The two one‐sided paired *t*‐test (TOST) method (Schuirmann, 1987), also referred to as equivalence testing, was used with a 10% bound (ratio of 0.9 to 1.1) for each nutrient (Batterham, Van Loo, Charlton, Cliff, & Okely, 2016). The two methods are considered equivalent when the 90% CI around the mean ratio (difference) falls within the specified bounds. Equivalence testing has the strength over traditional testing of differences between means of allowing a priori designation of what is a clinical significant difference, although its use in dietary studies is limited. Although it has been suggested that an equivalence bound of 10% or 15% of the difference between methods is reasonable for nutrient intake comparisons (Batterham et al., 2016; Tugault‐Lafleur, Black, & Barr, 2017), one dietary intake validation study has used a bound of 20% (Thompson et al., 2015). Given uncertainties about what the acceptable bound should be, and what could be detected with our sample size, we also considered wider bounds of 15% and 20%, which have been used in other dietary validation contexts (Thompson et al., 2015; Tugault‐Lafleur et al., 2017). Analyses were performed separately for the two age groups (10–11 and 12–14 years).

Individual errors were described by calculating the percentages of children over and underestimating energy intake by categories of percent error. Bland–Altman plots were used to depict the individual differences in energy intake by the two methods (recall minus observed) versus the average energy intake by the two methods. Limits of agreement (LOA) were calculated as the mean difference ±1.96 standard deviations, and LOA lines were depicted on the figures. Data were log‐transformed, and the differences between log‐transformed energy intakes by the two methods are interpreted as ratios.

Three sources of error were examined: memory, portion estimation and use of standard recipes. Errors in memory were assessed by counting the number of omissions (foods consumed not reported) and intrusions (foods reported that were not consumed) from the 24HR for each adolescent. The numbers and types of foods were described. Errors in portion estimation were assessed by comparing the reported food amounts by 24HR to the observed amounts by OWR for the most commonly consumed foods. We considered foods that had at least 60 consumption episodes across all children that were present in both OWR and 24HR, as this captured most frequently consumed foods, separated out errors of omission or intrusions and was sufficient to detect equivalence with a 25% bound. A wider bound of acceptability was selected for equivalence testing of individual food portions than for total nutrient intakes. Equivalence testing was conducted using mixed linear regression models with repeated measures to account for multiple consumption episodes per child. Errors due to the use of standard recipes versus household specific recipes were assessed in two ways. First, we compared the median nutrient intakes of children with household recipes (recipes were observed in 109 households) to their intakes with standard recipes replacing household recipes using Wilcoxon signed‐rank tests. Second, we compared mean nutrient intakes of all children (with or without observed household recipes) by OWR with standard recipes replacing household recipes to intakes by 24HR with standard recipes using TOST (same as with the primary outcome analyses). We also repeated this final analysis limiting to the adolescents from the 109 households where recipes were observed.

The prevalence of nutrient intake inadequacy was estimated separately from OWR and 24HR for each age group using the NCI method to estimate population usual intake distributions using both days of dietary information adjusting for within‐person variation (Tooze et al., 2010). The NCI method consists of a series of SAS macros to model usual intake (MIXTRAN) and estimate population distributions of usual intake (DISTRIB). The MIXTRAN macro estimated usual intakes using a one‐part mixed linear model to estimate within‐ and between‐person variation in nutrient intakes, removes the within‐person variation and estimates amounts of nutrient intakes. All children were included in the model rather than running a separate model for each age group, which allowed for pooling of variance as recommended when the number of second day intakes is less than 50 per subgroup (Kirkpatrick, Subar, & Tooze, 2017; National Cancer Institute, 2011). However, we included age group as a covariate to allow for the estimation of separate usual intake distributions for each age group by the DISTRIB macro, which uses the modelled intakes from MIXTRAN to perform Monte Carlo simulations to construct a population usual intake distribution of nutrient intakes and estimate the proportion of the population with intakes below the Estimated Average Requirement (EAR). The EAR cutpoints method was used to assess age‐group level nutrient intake adequacy (Institute of Medicine, 2000). EAR values were from the U.S. Institute of Medicine for males and females ages 9–13 years given that global EAR are not available (Institute of Medicine, 2006). The EAR for iron was adjusted for 10% bioavailability from the IOM value of 5.9 mg/day assuming 18% bioavailability (Institute of Medicine, 2002). The EAR for zinc was from IZiNCG for an unrefined diet (International Zinc Nutrition Consultative Group [IZiNCG] et al., 2004). Although there is no established cut‐off for high risk of dietary inadequacy for public health, we consider 25% as high risk as this has previously been used to define high risk of inadequate zinc intake (Wessells & Brown, 2012).

## 3 RESULTS

A final sample of 237 children was included in the study. The response rate was 95%. The majority of children (86%) in our sample attended school (Table S1). Of those attending school, 48% of younger and 54% of older children attended public school. Among all children, 59% of their mothers and 68% of fathers had at least some schooling.

Nutrient intakes calculated from 24HR were lower than from OWR (Table 1). Mean energy intakes by recall were 1,717 and 1,848 kcal, and from OWR were 1,919 and 1,975 kcal, for 10–11 and 12–14 years, respectively. Relative differences were assessed by ratios of log‐transformed intakes from 24HR to OWR (Table 2). The only nutrient found equivalent at the 10% bound was fat for 12–14‐year‐olds, with a mean relative difference of 0.98 (90% CI [0.91, 1.06]) or 2% error. Although the energy intake ratio for the older children was 0.92 (or 8% error), the CI did not fall within the 10% bound for equivalence. This was also the case for many other nutrients. If we used wider bounds of acceptability, for older children, 10 of the 15 nutrients including energy would fall within a 15% bound (defined as 90% CI [0.85, 1.15]), and all nutrients except vitamin B12 would fall within 20% bound (defined as 90% CI [0.80, 1.20]). For 10–11‐year‐olds, ratios for iron, vitamin C, riboflavin and vitamin B6 would have been equivalent within a 15% bound, whereas many nutrient ratios were marginally close to equivalence at the 15% (energy, protein, calcium and vitamin A). All nutrients except folate and vitamin B12 would have been acceptable within a 20% bound for the younger children.

**TABLE 1:** Nutrient intakes of children by 1 day of observed weighed records and 24‐h recall conducted

	Observed weighed records				24‐h recall			
	Mean	(SD)	Geometric mean	Median	Mean	(SD)	Geometric mean	Median
Ages 10–11 years (*n* = 132)								
Energy (kcal)	1,919	(549)	1,847	1,830	1,717	(605)	1,630	1,647
Protein (g)	52.0	(19.9)	48.6	48.3	46.7	(21.2)	43.1	41.2
Fat (g)	52.9	(21.4)	48.8	49.7	46.8	(21.1)	42.9	42.9
Carbohydrate (g)	301	(92)	288	288	270	(103)	253	262
Calcium (mg)	366	(221)	315	317	344	(247)	283	266
Iron (mg)	12.4	(5.0)	11.5	11.4	11.2	(4.8)	10.3	10.4
Zinc (mg)	7.0	(2.9)	6.5	6.3	6.2	(2.9)	5.8	5.6
Vitamin A (mcg RAE)	388	(281)	300	283	334	(255)	277	266
Vitamin C (mg)	75	(54)	58	61	74	(73)	57	60
Thiamin (mg)	0.73	(0.28)	0.68	0.69	0.64	(0.28)	0.59	0.56
Riboflavin (mg)	0.71	(0.42)	0.62	0.62	0.67	(0.43)	0.57	0.56
Niacin (mg)	11.7	(5.7)	10.4	10.5	10.4	(6.2)	9.2	8.6
Vitamin B6 (mg)	1.2	(0.5)	1.1	1.1	1.0	(0.5)	1.0	1.0
Folate (mcg DFE)	227	(130)	200	193	189	(101)	169	162
Vitamin B12 (mcg)	1.3	(1.6)	0.6	0.9	1.2	(1.3)	0.6	0.7
Ages 12–14 years (*n* = 105)								
Energy (kcal)	1,975	(629)	1,869	2,003	1,848	(802)	1,724	1,611
Protein (g)	51.6	(18.1)	48.3	51.1	48.1	(21.1)	44.5	43.8
Fat (g)	50.5	(23.6)	45.1	45.3	49.4	(24.8)	44.2	44.0
Carbohydrate (g)	319	(108)	300	312	295	(143)	271	271
Calcium (mg)	364	(233)	311	296	344	(258)	285	248
Iron (mg)	12.6	(5.1)	11.6	12.4	11.8	(5.7)	10.8	10.1
Zinc (mg)	7.1	(2.7)	6.5	6.9	6.6	(3.1)	6.0	6.0
Vitamin A (mcg RAE)	440	(416)	316	307	362	(255)	294	274
Vitamin C (mg)	79	(54)	61	69	74	(58)	56	62
Thiamin (mg)	0.74	(0.31)	0.68	0.69	0.65	(0.28)	0.60	0.58
Riboflavin (mg)	0.66	(0.33)	0.59	0.59	0.62	(0.34)	0.55	0.51
Niacin (mg)	11.0	(5.6)	9.7	10.1	10.5	(6.7)	9.1	8.5
Vitamin B6 (mg)	1.1	(0.5)	1.0	1.1	1.1	(0.5)	1.0	0.9
Folate (mcg DFE)	239	(137)	206	206	211	(133)	182	184
Vitamin B12 (mcg)	1.0	(1.3)	0.4	0.6	1.0	(1.2)	0.4	0.5

**TABLE 2:** Equivalence testing of ratios of nutrient intakes on 1 day by 24‐h dietary recall and observed weighed food records

	Ratio^a^ (24HR/OWR)	90% CI for test of equivalence of ratio		Equivalence		
	Mean	Lower	Upper	10% bound	15% bound	20% bound
Ages 10–11 years (*n* = 132)						
Energy (kcal)	0.88	0.85	0.92	‐	‐	Yes
Protein (g)	0.89	0.84	0.93	‐	‐	Yes
Fat (g)	0.88	0.82	0.94	‐	‐	Yes
Carbohydrate (g)	0.88	0.84	0.92	‐	‐	Yes
Calcium (mg)	0.90	0.84	0.96	‐	‐	Yes
Iron (mg)	0.89	0.85	0.93	‐	Yes	Yes
Zinc (mg)	0.88	0.84	0.93	‐	‐	Yes
Vitamin A (mcg RAE)	0.92	0.83	1.02	‐	‐	Yes
Vitamin C (mg)	0.98	0.89	1.07	‐	Yes	Yes
Thiamin (mg)	0.88	0.83	0.92	‐	‐	Yes
Riboflavin (mg)	0.93	0.87	0.98	‐	Yes	Yes
Niacin (mg)	0.88	0.83	0.94	‐	‐	Yes
Vitamin B6 (mg)	0.90	0.86	0.95	‐	Yes	Yes
Folate (mcg DFE)	0.84	0.79	0.90	‐	‐	‐
Vitamin B12 (mcg)	0.88	0.74	1.03	‐	‐	‐
Ages 12–14 years (*n* = 105)						
Energy (kcal)	0.92	0.87	0.98	‐	Yes	Yes
Protein (g)	0.92	0.86	0.99	‐	Yes	Yes
Fat (g)	0.98	0.91	1.06	Yes	Yes	Yes
Carbohydrate (g)	0.90	0.85	0.95	‐	Yes	Yes
Calcium (mg)	0.92	0.85	0.99	‐	Yes	Yes
Iron (mg)	0.93	0.87	0.99	‐	Yes	Yes
Zinc (mg)	0.92	0.86	0.98	‐	Yes	Yes
Vitamin A (mcg RAE)	0.93	0.84	1.03	‐	‐	Yes
Vitamin C (mg)	0.92	0.82	1.04	‐	‐	Yes
Thiamin (mg)	0.89	0.84	0.95	‐	‐	Yes
Riboflavin (mg)	0.93	0.87	1.00	‐	Yes	Yes
Niacin (mg)	0.93	0.87	1.01	‐	Yes	Yes
Vitamin B6 (mg)	0.95	0.89	1.02	‐	Yes	Yes
Folate (mcg DFE)	0.88	0.83	0.95	‐	‐	Yes
Vitamin B12 (mcg)	1.02	0.82	1.26	‐	‐	‐

*Note*: (1 − ratio) * 100 is equal to the percent error, and ratios between 0.9 and 1.1 are equivalent to a 10% bound around the mean percent error. A 90% confidence interval is used because two one‐sided tests are performed (each with *α* of 0.05).

a The ratio is back‐transformed from the difference in the log‐recalled nutrient minus the log‐observed nutrient intake.

The median percentage of estimated energy intakes from major food groupings was generally similar from both methods. Only energy intake from two out of 11 food groups were significantly different between the two methods (Table 3). The percent of energy from cereals was underestimated by older children (*p* < 0.04). Condiments (spices) were a very minor contributor to energy by both methods (1% or less) but overestimated by recall for both younger (*p* = 0.01) and older (*p* = 0.003) children.

**TABLE 3:** Percent of energy intake from major food groups on 1 day by 24‐h dietary recall and observed weighed food records

	Observed weighed records Median	25th, 75th percentiles	24‐h recall Median	25th, 75th percentiles	*p* value from Wilcoxon sign‐rank test
Ages 10–11 years					
Cereals	59.4	46.6, 67.7	56.6	45.7, 67.3	0.13
Roots and tubers	0	0, 0	0	0, 0	0.13
Beans	0	0, 4.1	0	0, 4.3	0.59
Nuts and seeds	0.7	0, 10.2	0	0, 11.2	0.92
Vegetables	2.6	1.6, 4.1	2.9	1.6, 4.5	0.25
Fruits	0	0, 1.7	0	0, 1.1	0.27
Animal source foods	2.9	0.6, 7.9	2.6	0.4, 8.1	0.98
Fats/oils	9.9	6.0, 15.6	10.9	6.2, 16.0	0.31
Sugars	0.9	0, 5.0	0	0, 5.2	0.54
Condiments	0.9	0.5, 1.4	1.1	0.6, 1.7	0.01
Beverages (non‐dairy)	2.7	0, 5.9	2.7	0, 7.3	0.22
Ages 12–14 years					
Cereals	61.6	51.5, 67.9	56.8	47.8, 68.7	0.04
Roots and tubers	0	0, 0	0	0, 0	0.44
Beans	0	0, 6.6	0	0, 5.7	0.24
Nuts and seeds	1.3	0, 9.0	0	0, 11.8	0.84
Vegetables	2.8	2.1, 4.2	3.2	2.0, 4.6	0.42
Fruits	0	0, 2.2	0	0, 1.3	0.27
Animal source foods	1.7	0, 5.3	1.9	0, 5.9	0.36
Fats/oils	11.0	6.1, 16.2	12.2	6.4, 17.1	0.10
Sugars	0	0, 2.9	0	0, 3.3	0.32
Condiments	1.0	0.5, 1.7	1.3	0.7, 1.9	0.003
Beverages (non‐dairy)	2.9	0, 5.9	2.4	0, 7.1	0.39

On an individual level, only 26% of younger and 17% of older children had energy intakes by recall within 10% of their OWR intakes (Table S2); 49% and 43%, respectively, had reported energy intakes within 20% error. Although overall both groups of children underestimated intakes, many individual children had very large overestimations. One child in the 12–14 years group overestimated energy intake by 190%. For sensitivity analysis, we tested equivalence after removing this child, and results were similar (e.g., mean energy intake ratio of 0.91 24HR: OWR; 90% CI [0.87, 0.96]); therefore, we included the child in all analyses. Bland–Altman plots (Figures S1 and S2) show wide LOA, with 95% of differences in energy intake expressed as a ratio for log‐transformed data of −0.71 to 0.46 and −0.77 and 0.61 for 10–11‐ and 12–14‐year‐olds, respectively. The differences in methods did not appear to vary by magnitude of energy intakes.

Errors in memory were assessed by the degree of agreement between the 24HR and OWR as to the number and type of food items. Twenty‐nine percent of younger and 28% of older children had perfect agreement. Fifty‐one percent of children of both ages were missing an entire food item on the 24HR (error of omission), and if we also included important distinguishing ingredients in mixed dishes, such as meat or carrots in a rice dish, then 56% of young and 61% of older children had an error of omission; 32% and 27% of younger and older children, respectively, reported a food on the recall that was not on the OWR (error of addition), and including distinguishing ingredients of mixed dishes, 36% of young and 32% of older children had an error of addition. The most frequently omitted foods were sweet or savoury snack foods (29% of all omitted foods), beverages (11% of foods) and fruits (11% of foods). Frequent additions included juices and sweet snacks such as cookies or cakes.

Although the overall direction in bias for portion size estimation by 24HR was underestimation, some foods were overestimated (Table S3). Among the cooked grain‐based foods, rice was underestimated by 1% to 6% depending on the form, but all forms were within 25% bound. Cakes and other sweet grain‐based snack foods were underestimated by 6% and within the 25% bound. On the other hand, portions of *To*, a maize‐based thick porridge, were overestimated by a mean of 22%. Fish was overestimated by 5% and meat underestimated by only 1% (both within 25% bound).

The impact of using standard recipes was assessed by comparing nutrient intakes from OWR using observed recipes to intakes from OWR with standard recipes replacing observed recipes among the 109 children with observed household recipes. With the exception of iron and riboflavin, there were no significant differences between median intakes for the assessed nutrients (*p* < 0.05). In addition, the absolute differences in iron and riboflavin between the two methods were negligible (0.1–0.3 mg). Among the entire sample of children, when we conducted the main analyses replacing observed recipes with standard recipes, the nutrient ratios were very similar to the main analyses that used observed recipes for OWR and the equivalence results were unchanged. Lastly, when the analyses were limited to adolescents for whom observed recipes were available, the results were similar.

Prevalences on inadequacy (POIs) were generally similar for both age groups (Table 4). POI by OWR was highest for calcium (100% for both age groups) and vitamin B12 (81% for 12–14‐year‐olds), and POI by 24HR was similar. POI by OWR was high (more than 25%) for eight out of the 11 micronutrients. Compared with the OWR estimates, the POI for all nutrients except vitamin C, calcium and vitamin B12 (for 12–14 years only) was overestimated. The nutrients with the greatest overestimation in POI by 24HR were vitamin A (32 to 35 percentage points [pp]) and niacin (30 pp for younger children). POI from 24HR were more than 25% for nine nutrients, and only niacin would be considered at high risk of inadequacy by 24HR but not by OWR. The percentage of total variation that was within‐person was more than 90% for vitamin A and C from 24HR (Table S4), whereas this was not the case for any nutrients by OWR. This very high within‐person variation once adjusted for using the NCI macros results in narrow population distributions of usual intake that are unstable and likely due to very large differences between intakes on the 2 days for some children. It should also be noted that the EAR cutpoint method does not perform well in cases when the POI is less than 5% or more than 95%, as was the case for these two nutrients (Institute of Medicine, 2000).

**TABLE 4:** Prevalence of inadequacy of micronutrient intakes estimated from observed weighed records and 24‐h recall

	EAR^a^	Ages 10–11 years			Ages 12–14 years		
		Observed weighed records	24‐h recall	Difference^b^	Observed weighed records	24‐h recall	Difference
Calcium (mg)	1,100	100	100	0	100	100	0
Iron (mg)	10.2/10.6	30	47	17	27	37	10
Zinc (mg)	7	52	75	23	52	67	15
Vitamin A (mcg RAE)	420/445	67	99	32	57	92	35
Vitamin C (mg)	39	4	<1	−4	2	0	−2
Thiamin (mg)	0.7	45	69	24	45	66	21
Riboflavin (mg)	0.8	78	82	4	77	82	5
Niacin (mg)	9	9	39	30	14	35	21
Vitamin B6 (mg)	0.8	7	19	12	8	14	6
Folate (mcg DFE)	250	66	84	18	63	78	15
Vitamin B12 (mcg)	1.5	67	72	5	81	80	−1

a The Estimated Average Requirements (EARs) were from IOM using values for males and females ages 9–13 years, with these exceptions: The EAR for iron was calculated from the IOM EAR of 5.7/5.9 mg for girls/boys adjusting for 10% bioavailability (IOM assumes 18% bioavailability). EAR for vitamin A are listed for girls/boys. The EAR for zinc was from IZINCG for unrefined diet. The same EARs were used for all age groups due to estimates being computed jointly for the entire population using the MIXTRAN macro of NCI method. The DISTRAN macro of NCI method was used to estimate separate distributions and prevalence of inadequacy for the two age subpopulations.

b Difference in prevalence of inadequacy using recall minus observation.

## 4 DISCUSSION

This validation study found that adolescents 10–14 years old systematically underestimated food intakes by 24‐h dietary recall. Energy intakes were underestimated on average by 8% by 12–14‐year‐olds and 12% for 10–11‐year‐olds. Equivalence was determined for most nutrients only with bounds of 15% (12–14‐year‐olds) or 20% (10–11‐year‐olds). As a result of underestimation of intakes by 24HR, micronutrient intake inadequacy was overestimated by 24HR, by as much as 30 pp or more for niacin and vitamin A.

Half of all children omitted a food in the 24HR and approximately one third of children added a food in the 24HR that was not observed. Errors in memory of a food consumed the previous day or reporting a food not consumed the previous day (but which may have been consumed on another day in the recent past or is a food typically consumed) are common in 24HR (Baxter et al., 2013; Ferguson, Gibson, & Opare‐Obisaw, 1994; Kerr, Wright, Dhaliwal, & Boushey, 2015). Suggestions for improvement in memory error in LMIC include providing the respondent with pictorial charts to mark foods as they are consumed, particularly for snacks and beverages (Gibson, Charrondiere, & Bell, 2017; Gibson & Ferguson, 2008), although one study that tested this did not find that it improved memory (Ferguson et al., 1995). Although the 24HR interview process specifically probes for commonly missed foods, it is unclear if and/or how much additional emphasis on this can reduce omission errors or possibly increase addition errors in children.

Errors in portion size estimation were generally biased towards underestimation and mainly responsible for overall underestimation of intake by 24HR. In fact, the mean energy intake of children with no errors of memory (no omissions or additions of foods in the 24HR) was 6% and 9% lower for the older and younger age groups, respectively, than intakes by OWR (data not shown), which is only slightly lower than the degree of underestimation overall (8% and 12%, respectively). Grain‐based foods were the highest source of energy in the children's diets, particularly rice that was underestimated by 1–6% by 24HR. Maize porridge (*To*) was overestimated, but although widely consumed, it contributed less energy than rice. Salted replicas were used to estimate both *To* and rice or rice dishes, which is recommended for main staple dishes (Gibson & Ferguson, 2008). In this population, play dough appeared to work well for estimating pieces of fish or meat with little error but not for larger items like sandwiches.

Other validation studies with adults in LMIC have reported problems with underestimation of main staples (Alemayehu et al., 2011; Ferguson et al., 1994; Ferguson et al., 1995). Adolescent girls in Mozambique underestimated weighed portions of staple grains (8% and 19% of maize porridge and rice, respectively) using photographs during one meal (Korkalo, Erkkola, Fidalgo, Nevalainen, & Mutanen, 2013). Some validation studies in children in HIC have concluded that use of two‐dimensional photographs of foods were better than three‐dimensional food models (Foster et al., 2008; Matheson, Hanson, McDonald, & Robinson, 2002). Pretesting of photos or any other methods of portion estimation should be conducted with adolescents in LMIC, particularly with staples or other widely consumed foods. Training adolescents in the use of selected portion estimation aids may also improve accuracy.

Underestimation of 24‐h intake has also been reported in validation studies among adults in LMIC using similar methodologies to the present study (Alemayehu et al., 2011; Ferguson et al., 1995; Gewa, Murphy, & Neumann, 2008). Based on reported mean energy intakes by OWR and 24HR from these published studies, ratios of intake by 24HR/OWR were estimated to be approximately 0.90 in Ethiopia (Alemayehu et al., 2011), 0.84 in Malawi (Ferguson et al., 1995) and 0.94 in Kenya (Gewa et al., 2008). In general, the degree of underestimation in adult women in LMIC was similar to that of the adolescents in the present study.

No validation studies of the 24HR method among adolescents in LMIC were found. Most validation studies with direct interview of children or adolescents in HIC focus on intake from a single meal (e.g., school lunch) rather than a 24‐h period (Appelhans et al., 2018; Foster et al., 2008; McPherson, Hoelscher, Alexander, Scanlon, & Serdula, 2000), use some estimate of energy expenditure as the validation standard (Burrows, Martin, & Collins, 2010; Livingstone et al., 2004) or have tested a self‐administered web‐based recall against an interviewer‐administered recall or energy expenditure (Baranowski et al., 2012; Bradley et al., 2016; Medin et al., 2017). One small validation study that compared recall to whole day OWRs among 11‐ to 15‐year‐old adolescents in the United States found that recalls overestimated energy intakes by about 10% (Kerr et al., 2015). Several review studies have been published on dietary assessment methods for school‐age children, but the variation in study designs (i.e., type of dietary method tested, type of validation standard, age of children, involvement of parent and time period of consumption) makes it difficult to surmise how accurately children can report recall overall a 24‐h period and make comparisons to the present study (McPherson et al., 2000; Tabacchi et al., 2013; Tugault‐Lafleur et al., 2017).

The results of our study show that adolescents underestimated their food intakes by 24HR, which resulted in underestimation of nutrient intakes and overestimation of the POI. The degree of underestimation was generally acceptable for 12–14‐year‐olds within a bound of 15%, and it is possible with a larger sample size of this older age group the CI would be narrower. However, the degree of error that is acceptable is debatable and should be carefully considered with regard to the purpose of the dietary information. For example, the 10% bound for this sample of 12‐ to 14‐year‐old adolescents would equate to approximately 187 kcal above or below the geometric mean intake of 1,869 kcal, whereas the 15% limit would equate to a ±280‐kcal acceptable bound. Although this is similar to or even better than some validation studies in adults, for which the 24HR method has been widely used for research and surveillance purposes, this amount of error may be considered unacceptable when a high degree of accuracy is desired.

The overestimation of POI when estimated using the adolescents' intakes by dietary recall is not surprising given the bias towards underestimation of nutrient intakes. The POI as estimated by the OWRs was quite high for the majority of micronutrients and largely above the threshold we would classify a nutrient deficiency as a public health problem; therefore, misclassification of dietary risk at the population level would not occur if using dietary recall in this population. However, for nutrients with low or moderate POI by OWR, misclassification can occur when POI by 24HR crosses a threshold of high risk (as was the case for niacin). When accurate POI is needed, 24HR may not be the method of choice, as was concluded by other validation studies (Alemayehu et al., 2011; Ferguson et al., 1995). 24HR should be sufficient for assessing general population risk level, changes over time, or comparing groups.

Our study has some limitations. First, there are methodological issues inherent to the dietary recall method that may contribute to differences in nutrient intakes from OWR. Conversion of amounts reported using volume or play dough to food amounts relies on conversion factors that are averages of collected factors or in some cases obtained from other studies or publications. The reliance on standard recipes for recalls is a source of potential error, although our testing did not conclude this was a major issue. There may have been errors introduced by differential assignment of food codes by the 24HR and OWR RA. Our methodology with OWR did not allow the RA to capture all household recipe preparation or to stay with the child for the full 24 h, although we accounted for the latter by matching the times of observation to the recalls and excluding food items reported on the recall that were consumed before or after the OWR RA was with the child. There were only six recalls with a reported food consumed after the OWR RA left the home and sensitivity analysis that included these foods did not have a meaningful impact on the results. Although full‐day observations could have influenced behaviour, we emphasized the purpose of the study to the adolescents and their caregivers and anecdotal reports by the RA did not suggest that eating behaviours were changed or improved by their presence due to limited resources of the study participants. Despite the prior day observation, the adolescents had a high rate of food omissions, and this could have been even greater without observation or potential heightened awareness of their food intake prior to the 24HR. There was some uncertainty about reported ages that may have resulted in some misclassification, but we used multiple sources of information where possible to classify children into the two age groupings.

Strengths of our study include foremost the direct observation and weighing of all foods and beverages consumed from the first to last meal of the day by accompanying the adolescent to school or other activities (i.e., market). In addition, the collection of data on recipes and average portion weights of foods sold by vendors on the school grounds enhanced accuracy of the intakes of foods consumed outside of the homes.

In conclusion, this validation study of adolescents in Burkina Faso, the dietary recall method could be used to assess population dietary risk levels, changes in intakes over time, and differences between groups, but its use remains questionable when a high level of accuracy is desired to estimate mean intakes or POI. More research should be conducted with adolescents in LMIC to determine whether error can be reduced by additional methods, such as training of adolescents to use pictorial charts to self‐record foods during the consumption period of interest and in use of portion estimation aids, and targeted probing for commonly omitted foods.

## CONFLICTS OF INTEREST

The authors declare that they have no conflicts of interest.

## CONTRIBUTIONS

JEA, MM, and DKO designed the research. JEA and MM conducted the research. JEA analysed data and drafted the manuscript. All authors read and approved the final manuscript.

## Supporting information

Table S1. Characteristics of adolescents
Table S2. Percent of children falling within ranges of percent error in estimating nutrient intakes by 24‐h recall compared to observed weighed records
Table S3. Comparison of food portion amounts estimated by 24‐h recall and observed weighed records †
Table S4. Usual intake distributions, variance estimates, and prevalence of inadequacy of micronutrient intakes estimated from direct observation and 24 h recall of children.
