# Supplementary material for: Validation of 24‐h dietary recall for estimating nutrient intakes and adequacy in adolescents in Burkina Faso
Source: Matern Child Nutr. 2020 Apr 26;16(4):e13014. doi: 10.1111/mcn.13014 (PMC7503205; doi:10.1111/mcn.13014)
Supplement: Supplementary file 1 — Table S1. Characteristics of adolescents Table S2. Percent of children falling within ranges of percent error in estimating nutrient intakes by 24‐h recall compared to observed weighed records Table S3. Comparison of food portion amounts estimated by 24‐h recall and observed weighed records † Table S4. Usual intake distributions, variance estimates, and prevalence of inadequacy of micronutrient intakes estimated from direct observation and 24 h recall of children. [file MCN-16-e13014-s001.pdf]

Supplementary information

Figure S1. Bland-Altman plot of log-difference (ratio) versus average of recall and observed energy intake of 10-11 year old children

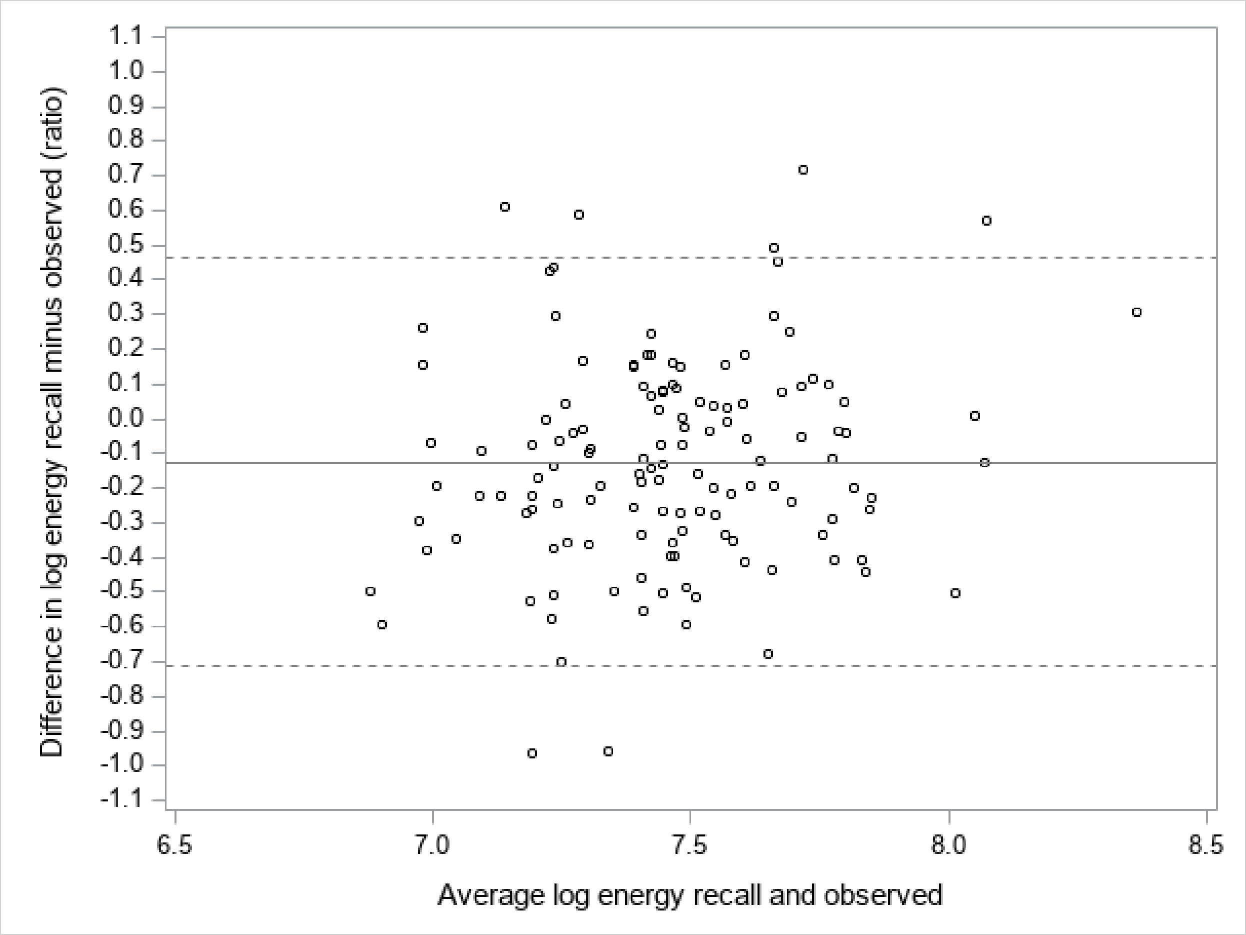

The grey solid horizontal line is at the mean difference in energy intake calculated as recall minus observed energy, expressed as a ratio for log-transformed data (mean ratio = -0.125). The grey dashed lines are the upper and lower lines of agreement (mean +/- 1.96 SD) containing 95% of values.

Figure S2. Bland-Altman plot of the log-difference (ratio) versus log average of recall and observed energy intake of 12-14 year old children

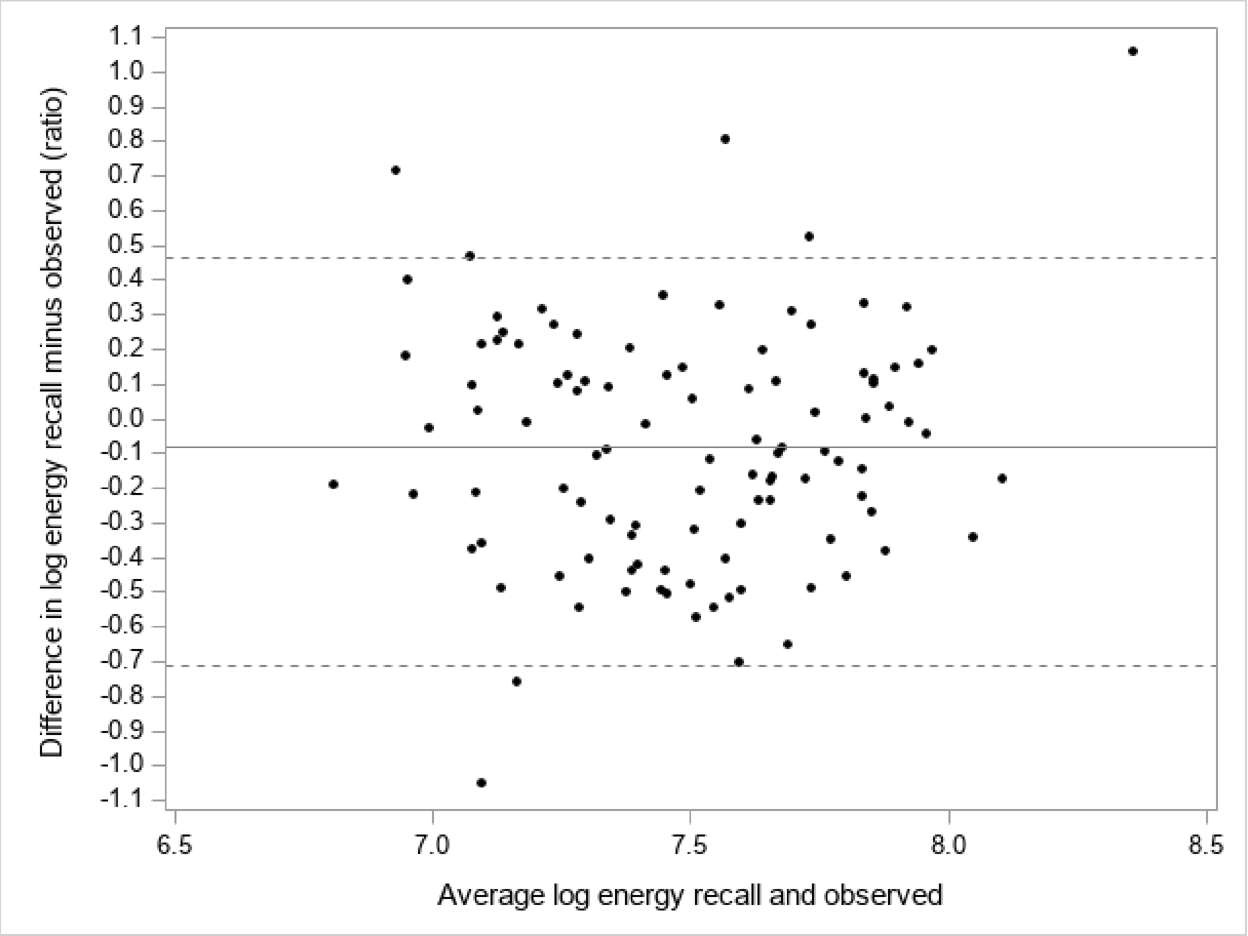

The grey solid horizontal line is at the mean difference in energy intake calculated as recall minus observed energy, expressed as a ratio for log-transformed data (mean ratio = -0.08). The grey dashed lines are the upper and lower lines of agreement (mean +/- 1.96 SD) containing 95% of values.

Table S1. Characteristics of adolescents

	Ages 10-11 years	Ages 12-14 years
Number	132	105
Sex		
Male	61 (46.2%)	51 (48.6%)
Female	71 (53.8%)	54 (51.4%)
School attendance		
None	12 (9.1%)	20 (19.1%)
Public primary school	58 (43.9%)	41 (39.2%)
Private primary school	62 (47.0%)	44 (41.9%)
Child education level if attending school		
Primary class 3	2 (1.7%)	0
Primary class 4	35 (29.2%)	11 (12.9%)
Primary class 5	44 (36.7%)	24 (28.2%)
Primary class 6	39 (32.5%)	50 (58.8%)
Maternal education level		
None	48 (36.4%)	48 (46.2%)
Primary (class 1-6)	34 (25.8%)	20 (19.2%)
Middle (class 7-10)	22 (16.7%)	15 (14.4%)
Secondary (class 11-13)	13 (9.9%)	5 (4.9%)
University	10 (7.6%)	10 (9.6%)
Other	3 (2.3%)	6 (5.8%)
Unknown	2 (1.5%)	0
Paternal education level		
None	33 (25.4%)	43 (41.0%)
Primary (class 1-6)	25 (19.2%)	13 (12.4%)
Middle (class 7-10)	17 (13.1%)	10 (9.5%)
Secondary (class 11-13)	12 (9.2%)	7 (6.7%)
University	30 (23.1%)	11 (10.5%)
Other	10 (7.7%)	18 (17.1%)
Unknown	3 (2.3%)	3 (2.9%)

Table S2. Percent of children falling within ranges of percent error in estimating nutrient intakes by 24-h recall compared to observed weighed records

	< - 50%	- 40.1 to -50%	-30.1 to -40%	-20.1 to -30%	-10.1 to -20%	<0 to -10%	>0 to +10%	+10.1 to +20%	+20.1 to +30%	+30.1 to +40%	+40.1 to +50%	> + 50%
Ages 10-11 y												
Energy	2.3	5.3	13.6	17.4	15.2	13.6	12.1	8.3	3.8	2.3	0	6.1
Protein	3.8	9.9	10.6	16.7	10.6	10.6	8.3	9.1	4.6	5.3	3.0	7.6
Fat	7.6	11.4	9.9	10.6	9.1	15.2	10.6	6.1	3.8	0.8	3.0	12.1
Carbohydrate	5.3	6.8	9.9	13.6	15.2	16.7	9.1	10.6	5.3	2.3	0.8	4.6
Calcium	5.3	12.1	8.3	12.9	13.6	11.4	10.6	7.6	1.5	0.8	3.0	12.9
Iron	2.3	9.1	7.6	15.9	16.7	14.4	9.1	7.6	9.1	0.8	3.8	3.8
Zinc	3.8	7.6	12.9	12.1	15.2	12.9	12.1	9.9	3.8	3.0	0.8	6.1
Vitamin A	15.2	6.1	10.6	9.9	9.9	9.1	9.9	3.8	3.8	2.3	1.5	18.2
Vitamin C	12.1	4.6	8.3	12.9	9.9	9.1	6.8	3.8	2.3	3.0	2.3	25.0
Thiamin	3.0	14.4	11.4	14.4	9.9	11.4	12.1	3.0	6.1	3.8	4.6	6.1
Riboflavin	5.3	8.3	8.3	15.2	10.6	12.9	10.6	9.1	2.3	2.3	1.5	13.6
Niacin	6.8	12.1	7.6	12.9	12.1	7.6	7.6	9.9	7.6	6.1	1.5	8.3
Vitamin B6	3.8	10.6	9.1	14.4	14.4	9.9	9.9	7.6	2.3	6.1	2.3	9.9
Folate	9.1	7.6	11.4	15.9	13.6	5.3	11.4	8.3	6.1	1.5	2.3	7.6
Vitamin B12	22.0	6.8	9.1	7.6	6.1	4.6	8.3	5.3	1.5	0.8	2.3	25.8
Ages 12-14y												
Energy	2.9	4.8	16.2	10.5	14.3	9.5	7.6	11.4	8.6	7.6	1.9	4.8
Protein	4.8	8.6	15.2	6.7	11.4	12.4	12.4	4.8	4.8	7.6	4.8	6.7
Fat	4.8	8.6	9.5	12.4	9.5	6.7	11.4	10.5	3.8	2.9	2.9	17.1
Carbohydrate	3.8	8.6	13.3	9.5	14.3	13.3	6.7	8.6	9.5	3.8	3.8	4.8
Calcium	7.6	11.4	9.5	8.6	15.2	7.6	4.8	11.4	4.8	1.9	5.7	11.4
Iron	5.7	4.8	17.1	8.6	10.5	9.5	13.3	7.6	3.8	4.8	4.8	9.5
Zinc	4.8	5.7	17.1	7.6	12.4	12.4	10.5	7.6	5.7	4.8	1.9	9.5
Vitamin A	15.2	1.9	8.6	7.6	13.1	7.6	9.5	4.8	6.7	5.7	1.0	18.1
Vitamin C	10.5	6.7	11.4	7.6	7.6	7.6	7.6	9.5	8.6	2.9	4.8	15.2

Thiamin	2.9	11.4	18.1	9.5	8.6	13.3	8.6	6.7	5.7	4.8	2.9	7.6
Riboflavin	7.6	5.7	7.6	12.4	14.3	9.5	4.8	12.4	9.5	2.9	1.9	10.5
Niacin	5.7	5.7	14.3	8.6	9.5	12.4	13.3	7.6	6.7	1.9	3.8	10.5
Vitamin B6	4.8	3.8	16.2	9.5	8.6	14.3	12.4	5.7	1.9	8.6	4.8	9.5
Folate	6.7	8.6	19.1	11.4	9.5	3.8	13.3	7.6	6.7	1.0	3.8	8.6
Vitamin B12	18.1	8.6	4.8	5.7	8.6	4.8	6.7	4.8	5.7	3.8	1.0	27.6

Table S3. Comparison of food portion amounts estimated by 24-h recall and observed weighed records †

	Portion estimation method for recall	Number of consumption episodes	Number of children	Geometric mean ratio (24HR/OWR)	90% CI Lower	90% CI Upper
To	Direct weight	129	125	1.22	1.13	1.31
Sauce with non-leafy vegetables	Volume	111	83	1.37	1.24	1.51
Peanut sauce	Volume	70	54	1.03	0.93	1.15‡
Rice	Direct weight	125	94	0.94	0.87	1.01‡
Rice with vegetables (Riz Gras)	Direct weight/volume	94	75	0.99	0.90	1.09‡
Rice with beans	Direct weight	64	57	0.94	0.83	1.06‡
Thin porridge	Volume	64	60	0.99	0.90	1.10‡
Sandwich	Playdough	97	85	0.60	0.55	0.66
Fish	Playdough	71	66	1.05	0.92	1.20‡
Meat	Playdough	62	48	0.99	0.87	1.14‡
Juice	Unit/volume	127	101	0.93	0.87	0.98‡
Cake/cookie/donut	Playdough	108	75	0.94	0.82	1.06‡

† Food portion amounts at each consumption episode that were reported on recalls and observed for weighed records were compared using a mixed model with the random effect of child. Gram amounts were log transformed, and the difference of logs is equivalent to the ratio of the recall to observed portion amounts. Foods that had at least 60 consumption episodes across all children were included, as this was sufficient to detect equivalence at a 25% bound.

‡ Equivalence at 25% bound

Table S4. Usual intake distributions, variance estimates, and prevalence of inadequacy of micronutrient intakes estimated from direct observation and 24h recall of children.

		Observed weighed record					24-h recall			
	EAR †	Distribution (25th, 50th, 75th percentiles)	Within person variance ‡		Prevalence of inadequacy		Distribution (25th, 50th, 75th percentiles)	Within person variance		Prevalence of inadequacy
			% of total	W/B				% of total	W/B	
Calcium, mg	1100		72.8	2.68				86.8	6.58	
10-11 y		286, 349, 424			100		283, 328, 379			100
12-14 y		297, 362, 438			100		295, 341, 393			100
Iron, mg	10.2/ 10.6		54.7	1.21				55.2	1.23	
10-11 y		10.1, 12.0, 14.2			30		8.9, 10.7, 12.8			47
12-14 y		10.2, 12.2, 14.4			27		9.5, 11.4, 13.6			37
Zinc, mg	7		76.4	3.24				69.1	2.24	
10-11 y		6.1, 6.9, 7.8			52		5.2, 6.1, 7.0			75
12-14 y		6.1, 6.9, 7.8			52		5.5, 6.4, 7.3			67
Vitamin A, mcg RAE	420/ 445		77.9	3.52				97.1	33.89	
10-11 y		288, 369, 467			67		310, 333, 358			99
12-14 y		318, 405, 509			57		346, 371, 398			92
Vitamin C, mg	39		82.2	4.62				93.2	13.70	
10-11 y		57, 70, 85			4		62, 70, 78			<1
12-14 y		61, 75, 90			2		63, 72, 80			0
Thiamin, mg	0.7		65.4	1.89				58.0	1.38	
10-11 y		0.62, 0.72, 0.84			45		0.53, 0.62, 0.73			69

12-14 y		0.61, 0.72, 0.83			45		0.54, 0.64, 0.75			66
Riboflavin, mg	0.8		80.6	4.15				71.5	2.51	
10-11 y		0.58, 0.68, 0.79			78		0.51, 0.62, 0.75			82
12-14 y		0.58, 0.68, 0.79			77		0.51, 0.62, 0.75			82
Niacin, mg	9		86.7	6.53				60.9	1.55	
10-11 y		10.1, 11.3, 12.7			9		8.0, 9.8, 12.0			39
12-14 y		9.6, 10.8, 12.1			14		8.2, 10.1, 12.3			35
Vitamin B6, mg	0.8		73.9	2.84				63.2	1.72	
10-11 y		1.0, 1.1, 1.3			7		0.8, 1.0, 1.2			19
12-14 y		0.9, 1.1, 1.3			8		0.9, 1.0, 1.2			14
Folate, mcg DFE	250		61.7	1.61				64.7	1.84	
10-11 y		176, 219, 272			66		153, 187, 228			84
12-14 y		181, 225, 279			63		165, 201, 244			78
Vitamin B12, mcg	1.5		50.1	1.00				65.8	1.92	
10-11 y		0.60, 1.07, 1.78			67		0.65, 1.04, 1.58			72
12-14 y		0.40, 0.76, 1.30			81		0.54, 0.88, 1.35			80

† The Estimated Average Requirements (EARs) were from IOM using values for males and females ages 9–13 years, with these exceptions: The EAR for iron was calculated from the IOM EAR of 5.7/5.9 mg for girls/boys adjusting for 10% bioavailability (IOM assumes 18% bioavailability). EAR for vitamin A are listed for girls/boys. The EAR for zinc was from IZINCG for unrefined diet.

‡ The variance components are computed for the entire population using the MIXTRAN macro of NCI method. The DISTRAN macro of NCI method was used to estimate separate distributions and prevalence of inadequacy for the two age subpopulations. W:B is the ratio of within to between-person variance.
